# An outbreak investigation of paediatric severe acute respiratory infections requiring admission to intensive care units – Fiji, May 2016

**DOI:** 10.5365/wpsar.2017.8.4.009

**Published:** 2018-06-21

**Authors:** Julie Collins, Viema Biaukula, Daniel Faktaufon, James Flint, Sam Fullman, Katri Jalava, Jimaima Kailawadoko, Angela Merianos, Eric Nilles, Katrina Roper, Meru Sheel, Mike Kama

**Affiliations:** a Hunter New England Population Health, Wallsend, Australia.; b National Centre for Epidemiology and Population Health, Australian National University, Canberra, Australia.; c Deployed by the Global Outbreak Alert and Response Network (GOARN), World Health Organization, Geneva, Switzerland.; d Division of Pacific Technical Support, World Health Organization, Suva, Fiji.; e Fiji Centre for Communicable Disease Control, Ministry of Health and Medical Services, Suva, Fiji.; f University of Helsinki, Helsinki, Finland.; g National Centre for Immunisation Research and Surveillance, Westmead, Australia.

## Abstract

**Introduction:**

Influenza-associated severe acute respiratory infections (SARI) are a major contributor to global morbidity and mortality. In response to a cluster of SARI cases and deaths in pregnant women, with two deceased cases testing positive for influenza A(H1N1)pdm09, an investigation was initiated to determine whether there was an increase of paediatric SARI cases admitted to divisional hospital intensive care units in Fiji in may 2016 compared to May 2013–2015.

**Methods:**

Retrospective case finding was conducted at the paediatric intensive care units (PICUs) in Fiji’s three divisional hospitals. Data were collected from 1 January 2013 to 26 May 2016. Cases were identified using a list of clinical diagnoses compatible with SARI.

**Results:**

A total of 632 cases of paediatric SARI with complete details were identified. The median age of cases was 6 months (Interquartile range: 2–14 months). Children aged less than 5 years had a higher rate of paediatric SARI requiring admission to a divisional hospital PICU in May 2016 compared to May 2013–2015 (Incidence rate ratio: 1.7 [95% CI: 1.1–2.6]). This increase was not observed in children aged 5–14 years. The case-fatality ratio was not significantly different in 2016 compared to previous years.

**Conclusion:**

The investigation enabled targeted public health response measures, including enhanced SARI surveillance at divisional hospitals and an emergency influenza vaccination campaign in the Northern Division.

## Introduction

Influenza-associated severe acute respiratory infections (SARI) are a major contributor to global morbidity and mortality, particularly among high-risk groups such as pregnant women and children. In 2008, the World Health Organization (WHO) estimated that there were 90 million new cases of seasonal influenza globally and 20 million cases of influenza-associated acute lower respiratory infections in children less than 5 years. (*1*) Influenza outbreaks typically occur during winter months in countries with temperate climates. In Pacific island countries, influenza outbreaks can occur throughout the year with less seasonal variation. (*2*) Influenza vaccination is an effective method for the prevention of influenza infection and subsequent complications. (*3*) WHO recommends influenza vaccination for pregnant women and children aged 6 months to 5 years to prevent severe disease requiring hospitalization. (*1*)

Fiji is a tropical archipelago in the South Pacific Ocean with an estimated population of 865 611. (*4*) National surveillance systems in Fiji capture information on influenza-like illness; however, surveillance for SARI is limited. (*5*) Fiji does not currently have a seasonal influenza vaccination policy; however, vaccination is recommended for high-risk groups including health-care workers, pregnant women, elderly persons and those with chronic illnesses. (*3*) Influenza vaccination is not publicly funded under Fiji’s national immunization programme, yet vaccines may be purchased privately from health-care providers. (*6*) Uptake of the influenza vaccine in Fiji has previously been reported as low. (*7*)

In May 2016, the Ministry of Health and Medical Services (MoHMS) in Fiji identified an increase in adult hospital admissions due to severe respiratory infections. In addition, a small cluster of pregnant women developed SARI, four of whom died. Two of the four deceased cases tested positive for the influenza A(H1N1)pdm09 virus. (*8*) In response to the increased SARI activity in adults, an investigation was conducted to determine if there was an increase in paediatric SARI cases requiring admission to divisional hospital paediatric intensive care units (PICUs) in Fiji in May 2016 compared to May 2013–2015 and to implement appropriate control measures. The investigation was led by the Fiji Centre for Communicable Disease Control (FCCDC) with support by WHO. This paper reports the findings of the investigation.

## Methods

We conducted retrospective case finding on 26–27 May 2016 at the three divisional hospital PICUs in Fiji: Colonial War Memorial Hospital (covering Central and Eastern Divisions), Labasa Divisional Hospital (Northern Division) and Lautoka Divisional Hospital (Western Division). Patient registers were reviewed to identify cases clinically compatible with SARI. Data from January 2013 to May 2016 were collected to ensure sufficient historical data to calculate baseline rates of disease.

A case-patient was defined as a child aged 0–14 years admitted to a divisional hospital PICU from 1 January 2013 to 26 May 2016 with any of the following diagnoses: pneumonia, severe pneumonia, acute respiratory distress syndrome, influenza, lower respiratory tract infection, upper respiratory tract infection or severe acute respiratory infection.

Data were collected on patients’ date of admission, age, diagnosis and outcome. Population data were calculated by applying estimated growth rates to 2007 Fiji census data. (*9*) Incidence rates were calculated for the month of May by division and paediatric age groups available from the census data (0–4 years, 5–9 years and 10–14 years). Incidence rate ratios (IRR) and Fisher’s exact 95% confidence intervals (CI) were calculated to compare incidence rates for May 2016 and May 2013–2015. The frequency and proportion of SARI cases were tabulated with a further breakdown of age for children less than 5 years (0–5 months, 6–11 months, 12–23 months, 24–35 months, 36–47 months and 48–59 months) as well as the 5–9 year and 10–14 year age categories. Case-fatality ratios (CFRs) were calculated for January–May 2016 and January–May 2013–2015. A Fisher’s exact two-sided p-value was calculated to compare the 2016 and baseline case-fatality ratios. The month of May 2016 in this paper refers to data collected up to 26 May 2016 (date of the investigation); data were collected and analysed for whole months in prior years. All analyses were conducted using Stata 14.1 (StataCorp LP, College Station, USA) and Microsoft Excel 2016 (Microsoft Corporation, Redmond, USA).

## Results

**Fig. 1:**
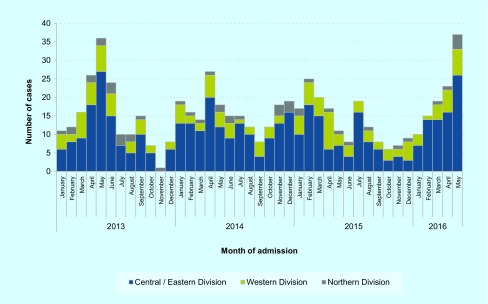
Cases of paediatric severe acute respiratory infection (SARI) admitted to divisional hospital paediatric intensive care units (PICUs) by division and month of admission, Fiji, January 2013 to May 2016 (*n* = 632)

We identified 632 cases of paediatric SARI with complete details requiring admission to divisional hospital PICUs between January 2013 and May 2016 (**Fig. 1**). The median age of paediatric SARI cases during the investigation period (January 2013–May 2016) was 6 months (Interquartile range: [IQR] 2–14 months). Ninety-three per cent (*n* = 586) of all cases identified during the investigation were in children aged less than 5 years. Moreover, 85% (*n* = 540) were in children aged less than 2 years.

**Fig. 1** shows the number of cases admitted by month and year of the investigation period. The rate of paediatric SARI in children less than 5 years was higher during the month of May 2016 when compared to the same period in 2013–2015 (IRR: 1.7 [95% CI: 1.1–2.6]) (**Table 1**). The rate increase in children less than 5 years was not statistically significant when stratified by division (**Table 1**).

**Table 1:** Incidence rate per 10 000 population and incidence rate ratio of paediatric SARI requiring admission to divisional hospital PICUs, Fiji, May 2016 and May 2013–2015

-	May 2016	May 2013–2015	-
	IR*	IR*	IRR (95% CI)^†^
**Central/Eastern Division**			
**0–4 years**	**5.6**	**3.5**	**1.6 (0.9–2.7)**
**5–9 years**	**0.3**	**0.2**	**1.5 (0.0–28.2)**
**10–14 years**	**0.2**	**-**	**-**
**Western Division**			
**0–4 years**	**2.2**	**1.3**	**1.7 (0.6–4.8)**
**5–9 years**	**-**	**0.2**	**-**
**10–14 years**	**-**	**-**	**-**
**Northern Division**			
**0–4 years**	**2.9**	**1.2**	**2.4 (0.5–11.2)**
**5–9 years**	**-**	**-**	**-**
**10–14 years**	**-**	**-**	**-**
**Divisions combined**			
**0–4 years**	**4.0**	**2.3**	**1.7 (1.1–2.6)**
**5–9 years**	**0.1**	**0.2**	**0.7 (0.0–7.5)**
**10–14 years**	**0.1**	**-**	**-**
**All years (0–14)**	**1.4**	**0.9**	**1.7 (1.1–2.6)**

* Monthly incidence rates were calculated for May in each year.

^†^ Incidence rate ratios could not be calculated for some groups because of zero counts.

CI: confidence interval, IR: incidence rate, and IRR: incidence rate ratio

The CFRs were not significantly different for cases of paediatric SARI requiring admission to divisional hospital PICUs in January–May 2016 (12.5%) compared to the same period in 2013–2015 (9.1%) (*P* = 0.343).

### Outbreak response

The FCCDC established enhanced SARI surveillance at divisional hospital PICUs to ensure continued monitoring. In addition, the FCCDC, with support from the WHO Emerging Diseases Clinical Assessment and Response Network, conducted critical care training with a particular focus on SARI for PICU staff in August 2016.

Paediatric SARI activity in the Central/Eastern and Western divisions had been increasing in the months before the investigation (**Fig. 1**). However, the increase appeared to be delayed in the Northern Division, allowing an opportunity to implement preventive measures. In anticipation of an increase in paediatric SARI cases in the Northern Division, the MoHMS and WHO Division of Pacific Technical Support facilitated a donation of 6000 doses of paediatric influenza vaccine. An emergency influenza vaccination campaign in the Northern Division was jointly coordinated by the Northern Division Public Health Team, the Fiji Expanded Programme on Immunization (EPI) and Labasa Divisional Hospital from July to September 2016. The vaccination campaign targeted children aged 6–12 months and achieved 84% coverage (Fiji EPI, unpublished data, 2016).

## Discussion

We found that children aged less than 5 years experienced a higher rate of SARI requiring admission to divisional hospital PICUs in the month of May 2016 compared to the same month in 2013–2015. The majority of SARI cases in the investigation period occurred in children aged less than 2 years (85%), which confirms that this age group is at a high risk of severe influenza-associated respiratory infections. (*1*, *10*)

Three months before the outbreak, Fiji was struck by one of the strongest tropical cyclones recorded in the southern hemisphere. Tropical Cyclone Winston resulted in 44 deaths and caused severe damage and displacement throughout Fiji. (*11*) Populations in crisis have a higher risk of outbreaks of acute respiratory infections, and this may have influenced the increase in paediatric SARI requiring PICU admissions among children aged less than 5 years in May 2016. (*12*)

The increased incidence of paediatric SARI in May 2016 may also have been influenced by circulating influenza A viruses. Influenza A was predominant in Fiji during April–May 2016, with A(H1N1)pdm09, A(H3) and some B viruses detected. (*13*) The April–May period began with more notifications of A(H1N1)pdm09; however, A(H3) was predominant overall. (*13*) Globally, the 2015–2016 influenza season was also marked by an early predominance of the influenza A(H1N1)pdm09 virus with influenza A(H3N2) predominant later in the global season. (*14*, *15*)

Several limitations were identified in the investigation. We only measured severe disease requiring admission to paediatric intensive care units; this paper does not provide a comprehensive estimate of paediatric SARI incidence. The investigation case definition was based on clinical diagnoses, which may have resulted in some misclassification of SARI cases. The etiology of SARI was not systematically investigated as suspected cases of influenza are not routinely confirmed by microbiological testing in Fiji; the assumption that the increase in paediatric SARI was due to influenza cannot be confirmed. CFRs should be interpreted in the context of PICU admissions rather than all paediatric SARI hospitalizations. Since the investigation was conducted in May 2016, CFRs were calculated for the period January–May for each year (2013–2016) and incidence rates for the month of May only (2013–2016). Small case numbers in some divisions and age groups may have influenced the results.

While recognizing there are competing priorities for health resources, the introduction of a seasonal influenza vaccination policy for high-risk groups, as per WHO recommendations, should be considered to address the ongoing burden of paediatric SARI in Fiji. (*1*, *3*)

## Conclusion

This investigation provided valuable information on the burden of paediatric SARI requiring admission to divisional hospital PICUs in Fiji in May 2016. The data were used to implement targeted public health response measures and enhance surveillance for paediatric SARI in divisional hospitals in Fiji.
